# Unraveling the mechanisms of LncRNAs in cervical cancer: a comprehensive review

**DOI:** 10.1038/s41420-025-02902-1

**Published:** 2025-12-13

**Authors:** Lusheng Liu, Zhongyu Han, Qian Qian, Xiaozhu Liu, Jing He, Di Wang, Jing Zhou, Guizhi Ma

**Affiliations:** 1 Department of Acupuncture and Moxibustion, Shanghai Traditional Chinese Medicine Integrated Hospital, Shanghai University of Traditional Chinese Medicine, Shanghai, China; 2 Shanghai Clinical Medical College of Integrated Traditional Chinese and Western Medicine, Shanghai University of Traditional Chinese Medicine, Shanghai, China; 3 Institute of Nephrology, Zhongda Hospital, Southeast University, Nanjing, China; 4 Rehabilitation Medicine Dept, Guilin Municipal Hospital of Traditional Chinese Medicine, Guilin, China; 5 The First Affiliated Hospital of Naval Medical University, Shanghai, China

**Keywords:** Cancer, Cancer immunotherapy

## Abstract

Cervical cancer (CC) ranks as the fourth most prevalent malignancy and the second leading cause of cancer-related mortality in women. Central to its pathology are long non-coding RNAs (LncRNAs), a class of transcripts exceeding 200 nucleotides that do not encode proteins. Instead, they function as critical regulators of gene transcription, chromatin remodeling, and cell cycle progression by interacting with DNA, RNA, or proteins, thereby influencing a range of physiological and pathological processes. As research on LncRNA function deepens, its critical role in tumor biology has become increasingly apparent. LncRNAs have attracted considerable attention in recent years regarding their role in the development and progression of CC. LncRNAs are involved in the proliferation, migration, and invasion of CC cells, and also regulate multiple signaling pathways by interacting with other molecules to influence tumor progression. Although several LncRNAs have been identified as biomarkers of CC, and research on their potential as therapeutic targets is advancing, their specific mechanisms of action and clinical application remain poorly understood. This review aims to comprehensively analyze the biological functions and mechanisms of LncRNAs in CC and explore their clinical application potential, providing new insights and directions for the early diagnosis and treatment of CC.

## FACTS

- LncRNAs play crucial roles in the development, progression, and therapeutic resistance of CC through diverse mechanisms, including the regulation of gene expression, epigenetic modifications, and modulation of signaling pathways.
- Through their interactions with DNA, RNA, or proteins, LncRNAs regulate fundamental biological processes in cervical cancer cells, including proliferation, apoptosis, invasion, and metastasis.
- Several LncRNAs are potential biomarkers for early CC diagnosis and prognosis, and their expression levels can be detected in various biological samples, including tissues, blood, and vaginal secretions.
- In cervical cancer, LncRNAs modulate pivotal signaling cascades, including the Wnt/β-catenin, MAPK, and PI3K/Akt pathways, which are integral to tumor progression and the development of drug resistance.
- Targeting LncRNAs or their interacting molecules offers promising therapeutic strategies for CC, including small molecule inhibitors, RNA interference technologies, and gene-editing tools such as CRISPR/Cas9.

## QUESTIONS

- What are the molecular mechanisms by which LncRNAs, through their interactions with DNA, RNA, and proteins, modulate the proliferation, apoptosis, invasion, and metastasis of cervical cancer cells?
- What are the specific mechanisms by which LncRNAs modulate key signaling pathways in CC, and how do these pathways contribute to tumor progression and therapeutic resistance?
- Which specific LncRNAs show promise as clinical biomarkers for the early diagnosis, prognostic assessment, and prediction of therapeutic response in cervical cancer, and what is their demonstrated value in these clinical applications?
- How can LncRNAs be targeted for therapeutic purposes in CC, and what are the potential advantages and challenges of using small molecules, RNA interference, or gene-editing technologies to modulate LncRNA function?
- What are the roles of LncRNAs in the tumor microenvironment of CC, and how do they influence immune cell infiltration, angiogenesis, and other microenvironmental factors that affect tumor progression and treatment outcomes?

## Introduction

Globally, cervical cancer (CC) represents the second most prevalent gynecological malignancy affecting women [1]. In low- and middle-income countries, CC presents a significant burden of morbidity and mortality, a situation driven predominantly by infections with high-risk human papillomavirus (HR-HPV) [2, 3]. Up to 99% cases of CC are linked to HPV, with HPV types 16 and 18 responsible for >70% of the cases [4, 5]. Current treatments for CC include surgery, radiotherapy, chemotherapy, and targeted therapy; however, patients with advanced and metastatic disease often experience limited efficacy owing to drug resistance and tumor metastasis [6, 7]. Early prevention is crucial, and vaccination remains an effective method for CC prevention [8]. However, in some developing countries, the incidence of CC has not decreased significantly because of socioeconomic factors, lack of prevention awareness, and imperfect vaccination programs [9, 10]. Therefore, an in-depth study of the pathological mechanisms of CC not only helps reveal the key molecular mechanisms underlying its development but also provides new strategies for early diagnosis and targeted treatment.

LncRNAs are RNA molecules with > 200 nucleotides that have been previously overlooked because of their inability to encode proteins [11]. Advances in high-throughput sequencing have illuminated the crucial function of LncRNAs in modulating gene expression through mechanisms such as epigenetic modifications, transcriptional regulation, and mRNA splicing [12, 13]. LncRNAs function as pivotal modulators of cellular growth, differentiation, and signaling, thereby underpinning the initiation and advancement of malignancies [14, 15]. In CC, LncRNAs modulate critical cellular processes, including proliferation, apoptosis, and invasion, via epigenetic and transcriptional control, while also being intimately involved in remodeling the immune microenvironment [16]. Additionally, LncRNAs show great potential in predicting prognosis and immunotherapy responses in patients with CC, and are used as biomarkers for early diagnosis and pathological classification [17]. This research uncovers novel targets, thereby providing a foundation for new strategies in the early diagnosis and targeted treatment of cervical cancer.

This review delineates the biological functions and regulatory mechanisms of LncRNAs in CC, examining their influence on cellular processes including proliferation, apoptosis, invasion, and migration. This study also reviews the potential clinical applications of LncRNAs in the diagnosis, treatment, and prognostic evaluation of CC while anticipating future research directions and challenges. As studies progress, LncRNAs are expected to become biomarkers and therapeutic targets for CC, thereby offering new approaches for early diagnosis and targeted treatment.

## LncRNAs are involved in the pathological mechanism of CC

### Characterization of LncRNAs

As non-protein-coding RNA molecules typically ranging from 200 to 100,000 nucleotides in length, LncRNAs fulfill essential biological functions by participating in key processes such as gene expression regulation, chromatin remodeling, cell cycle control, and cellular differentiation [18, 19]. Research indicates that while over 90% of the human genome is transcribed, protein-coding transcripts account for only about 3% of the total. The remainder, consisting largely of non-coding RNAs, serves a crucial function in maintaining stable protein expression within cells [20, 21].

The transcription of LncRNAs is typically mediated by RNA polymerase II and is governed by a variety of transcription factors [22]. The classification of LncRNAs into several categories, such as antisense, intronic, and promoter-associated, is determined by their location within the genome [23]. Although LncRNAs have relatively low transcription and splicing efficiencies and generally exhibit lower sequence conservation than mRNAs, their expression levels are also lower. However, LncRNAs often display strong tissue-specific expression patterns in cells [24].

**Fig. 1:**
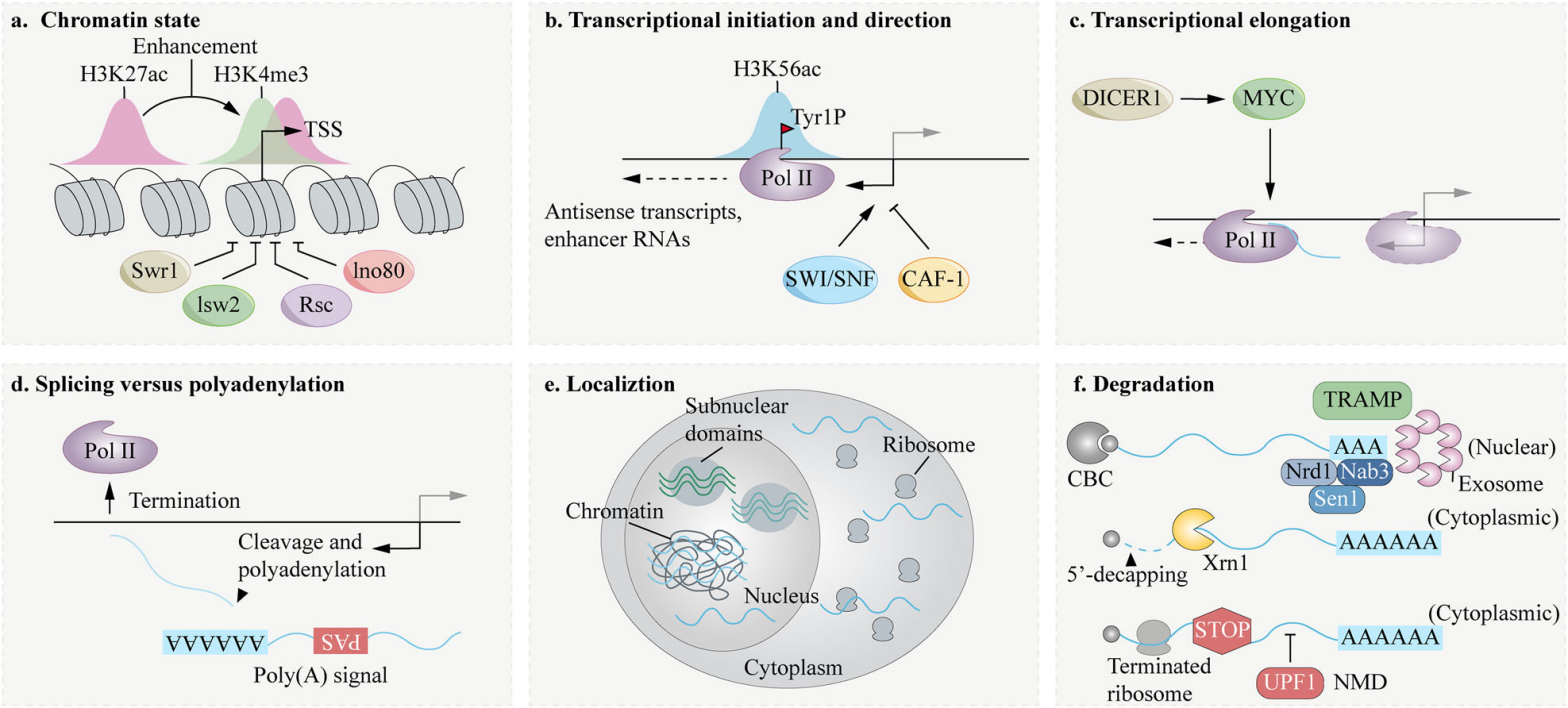
The active life cycle of long non-coding RNAs (lncRNAs) -birth, survival, and death. **a** Chromatin environment. **a** The transcription of lncRNAs is associated with a distinct chromatin signature. Their promoters are often marked by active enhancer and promoter-associated histone modifications, such as histone H3 lysine 4 trimethylation (H3K4me3) and H3 lysine 27 acetylation (H3K27ac). The chromatin remodeling complexes Swr1, Isw2, and Ino80 are implicated in modulating the chromatin landscape at these sites, potentially influencing lncRNA transcriptional accessibility. TSS, transcription start site. **b** Transcriptional initiation and direction. Initiation of lncRNA transcription is characterized by the phosphorylation of tyrosine 1 on the RNA Polymerase II (Pol II) C-terminal domain (CTD) and enrichment of histone marks like H3 lysine 56 acetylation (H3K56ac), particularly for antisense transcripts. This process, which often yields antisense transcripts and enhancer RNAs (eRNAs), is promoted by the SWI/SNF chromatin remodeling complex but is antagonized by the chromatin assembly factor CAF-1. **c** Transcriptional elongation. The elongation phase of lncRNA transcription is regulated by factors such as DICER1 and the oncoprotein MYC, which can influence the progression and stability of RNA Polymerase II along the gene body. **d** Splicing versus polyadenylation. Bidirectional promoters often lead to the production of a protein-coding mRNA in the cis-orientation and a lncRNA in the trans-orientation. The positioning of U1 splicing signals and poly(A) signals favors the splicing and maturation of the cis-oriented mRNA, while the trans-oriented lncRNA transcript is more likely to undergo early cleavage and polyadenylation, limiting its length and potential to be processed as a mRNA. **e** Localization. LncRNAs exhibit diverse subcellular localizations, which are closely linked to their functional mechanisms. They can be found associated with chromatin, within specific subnuclear domains (e.g., nucleoli, nuclear speckles), in the nucleoplasm, or exported to the cytoplasm. **f** Degradation. Many lncRNAs are rapidly turned over. Nuclear surveillance pathways, involving complexes such as TRAMP and the nuclear exosome, target and degrade aberrant or transient transcripts. Key players in nuclear termination and degradation include the Nrd1-Nab3-Sen1 complex. In the cytoplasm, lncRNAs can be subjected to degradation pathways typically associated with mRNAs, including 5’ decapping followed by exonucleolytic digestion by Xrn1, or nonsense-mediated decay (NMD). CBC, cap-binding complex.

In the nucleus, LncRNAs help organize and modify chromatin by interacting with DNA, RNA, and proteins, while regulating gene transcription processes [25]. In the cytoplasm, LncRNAs play a role in post-transcriptional regulation by controlling mRNA stability, translational efficiency, and coordinating protein processing events [26]. Studies have shown that LncRNA transcription can directly affect the expression of downstream genes, with either positive or negative regulatory effects [27]. LncRNAs exhibit significant differences in expression between tumors and healthy cells and are closely linked to carcinogenesis. They are rapidly emerging as key foci of oncology research in biological and medical fields [28, 29]. Additionally, LncRNAs exhibit potential regulatory functions in various tumors and are expected to serve as valuable sources for new biomarkers and drug targets [30] (Fig. 1).

### Regulation of CC cell proliferation by LncRNAs

LncRNAs play a crucial regulatory role in CC cell proliferation through complex and diverse mechanisms, including cell cycle regulation, transcription factor regulation, microRNA (miRNA) sponge effect, epigenetic modifications, and apoptosis inhibition. Collectively, these mechanisms drive the aberrant proliferation of CC cells and tumor progression (Table 1).

**Table 1:** Expression of relevant LncRNAs in CC.

LncRNA Type	Expression	Targeting	Function	Ref
MALAT1	Increased	caspase-3, caspase-8, Bax, Bcl-2 and Bcl-xL	Regulating the cell cycle to inhibit apoptosis, thereby promoting cell proliferation and invasion	[130]
		miR-124/RBG2 axis	Promoting the growth and invasion of HR-HPV (+) cancer cells	[131]
		miR-145	Promoting radiotherapy resistance	[132]
		miR-202-3p/periostin axis	Enhancing cell viability and promoting cell proliferation and metastasis	[133]
		miR-429	Promoting cell proliferation and invasion	[134]
		miR-625-5p/NF-κB axis	Promoting cell proliferation and invasion	[33]
		BRWD1 and PI3K/AKT signaling pathway	Promoting cisplatin resistance	[135]
HOTAIR	Increased	miR-143-3p/BCL2 axis	Promoting cell proliferation	[46]
		VEGF, MMP-9	Promoting cell invasion and EMT	[127]
		Notch signaling pathway	Promoting cell proliferation, invasion, and EMT	[136]
NEAT1	Increased	miR-124/NF-κB axis	Promoting cell invasion and metastasis	[137]
		miR-34a/LDHA-glycolysis axis	Promoting resistance to 5-Fu chemotherapy	[138]
H19	Increased	miR-138-5p/SIRT1 axis	Promoting cell proliferation and lymph node metastasis	[139]
CCAT2	Increased	Wnt/β-catenin signaling pathway	Promoting cell metastasis and increasing the recurrence rate of CC	[140]
ANRIL	Increased	PI3K/Akt signaling pathway	Promoting cell metastasis	[141]
PVT1	Increased	miR-503/ARL2 axis	Promoting cell proliferation, invasion, and metastasis	[142]
		EZH2/H3K27me3/miR-195	Enhancing PTX resistance and promoting cell EMT	[98]
TUG1	Increased	Bcl-2, caspase-3	Causing poor differentiation of CC and promoting cell EMT and metastasis	[143]
		MAPK signaling pathway	Promoting cisplatin resistance	[144]
AATBC	Increased	miR-1245b-5p	Promoting lymph node metastasis of cells	[145]
LINC01012	Increased	CDKN2D (p14ARF or p16INK4a)	Promoting cell proliferation and metastasis	[146]
LINC00511	Increased	miR-497-5p/MAPK1 axis	Promoting cell proliferation, invasion, and metastasis	[147]
LINC01287	Increased	miR-513a-5p/SERP1 axis	Promoting cell proliferation and metastasis while inhibiting apoptosis	[148]
FLVCR1-AS1	Increased	miR-381-3p/MAGT1 axis	Promoting cell proliferation, invasion, and metastasis; inhibiting apoptosis	[149]
		miR-23a-5p/SLC7A11 axis	Promoting cell proliferation, invasion, migration, and EMT; accelerating apoptosis	[150]
LINC00649	Increased	miR-216a-3p	Promoting cell proliferation, invasion, and metastasis, thereby accelerating CC progression	[151]
HOXA-AS3	Increased	miR-29a-3p	Promoting cell proliferation, invasion, and metastasis	[152]
TDRG1	Increased	miR-326/MAPK1 axis	Promoting cell proliferation, invasion, and metastasis	[153]
		miR-214-5p/SOX4	Promoting cell proliferation, invasion, metastasis, and EMT	[154]
		miR-330-5p/ELK1	Promoting cell proliferation and metastasis	[155]
FENDRR	Decreased	miR-15a/b-5p/TUBA1A axis	Inhibiting cell growth and metastasis	[156]
LAMTOR5-AS1	Decreased	miR-210-3p	Inhibiting the malignant transformation of cells	[157]
GAS5	Decreased	miR-424	Inhibiting cell proliferation, invasion, and lymph node metastasis	[158]
		miR-21, phosphorylation of Akt	Inhibiting cisplatin resistance	[104]
		miR-21/STAT3, TIMP3, PDCD4	Inhibiting cisplatin resistance	[159]
ENP1	Decreased	miR-27a-3p/EGR1	Inhibiting cell proliferation, motility, and EMT	[160]
FAM13A-AS1	Decreased	miRNA-205-3p/DDI2 axis	Inhibiting cell proliferation, invasion, and migration	[161]
MCM3AP-AS1	Decreased	miR-93	Inhibiting cell proliferation	[162]
PGM5-AS1	Decreased	miR-4284/DCN	Inhibiting cell proliferation, invasion, and migration	[163]
MEG3	Decreased	miR-21-5p	Inhibiting cell growth and metastasis	[164]
		miR-7-5p/STC1/ERs	Promoting apoptosis	[165]
		P-STAT3	Inhibiting cell proliferation while promoting apoptosis	[166]

*5-Fu* 5-fluorouracil, *ANRIL* Antisense Noncoding RNA in the INK4 Locus, *CC* cervical cancer, *CCAT2* colon cancer-associated transcript2, *EMT* epithelial–mesenchymal transition, *ERs* endoplasmic reticulum stress, *FENDRR* fetal-lethal non-coding developmental regulatory RNA, *FLVCR1-AS1* FLVCR heme transporter 1 antisense RNA 1, *GAS5* growth arrest-specific transcript 5, *HOTAIR* HOX transcript antisense RNA, *HOXA-AS3* HOXA cluster antisense RNA 3, *LncRNA* Long noncoding RNA, *MALAT1* Metastasis-associated lung adenocarcinoma transcript 1, *MCM3AP-AS1* Minichromosome Maintenance Complex Component 3 Associated Protein Antisense 1 MEG3: Maternally Expressed Gene, *MMP-9* matrix metalloproteinase-9, *NEAT1* Nuclear Enriched Abundant Transcript 1, *PTX* paclitaxel, *PVT1* plasmacytoma variant translocation 1 gene, *TDRG1* Testis developmental related gene 1, *TUG1* taurine-upregulated gene 1, *VEGF* vascular endothelial growth factor.

Several studies demonstrated that LncRNAs regulate cell cycle progression through various mechanisms, thereby promoting CC cell proliferation and tumor progression. For example, LncRNA NEAT1 is highly expressed in CC and promotes cell cycle progression, proliferation, and invasion of CC cells by upregulating cyclin D1 and CDK4, inhibiting caspase 3 activity, and activating the p-AKT, p-PI3K, and MMP2 signaling pathways [31]. Similarly, LncRNA MALAT1 is highly expressed in CC cells; however, it is undetectable in normal cervical tissues, where it promotes proliferation by upregulating cyclin D1, cyclin E, and CDK6 [32, 33]. Furthermore, LncRNA DLEU2 inhibits Notch signaling activity by downregulating p21 and p53 expression, thereby promoting CC cell proliferation [34]. This suggests that LncRNAs can shorten the cell cycle via various mechanisms, thereby promoting CC cell proliferation and tumor progression.

**Fig. 2:**
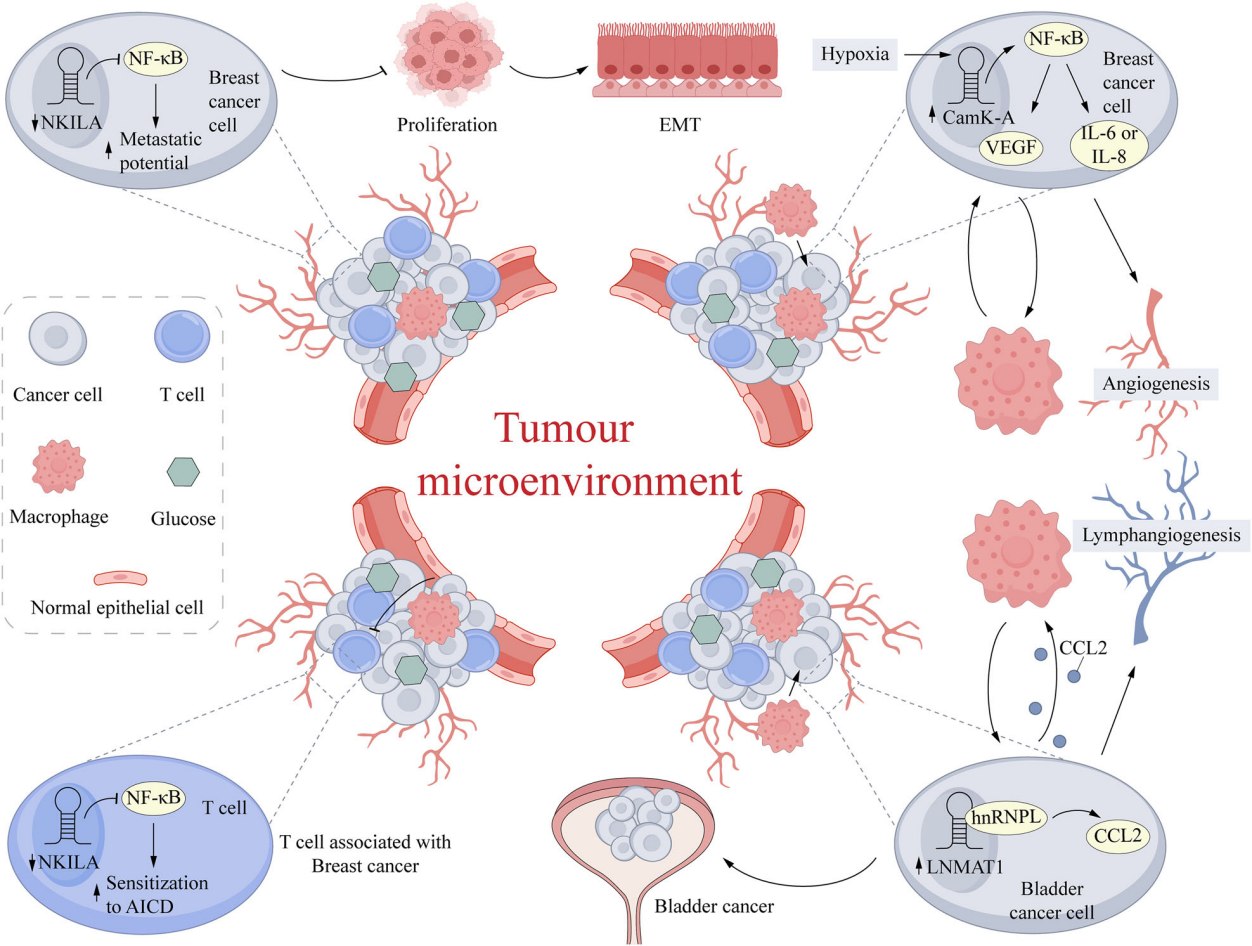
Long non-coding RNAs (lncRNAs) related signaling pathways in cervical cancer. **a** **Hippo Signaling Pathway:** Pathway activity is initiated by upstream membrane proteins such as FAT1-4. Signals are transduced through adapter proteins like FRMD6, Merlin, and KIBRA, activating a kinase cascade consisting of MST1/2 with SAV1, and LATS1/2 with MOB1. Activated LATS1/2 phosphorylates the downstream effectors YAP/TAZ, leading to their cytoplasmic retention by 14-3-3 proteins and subsequent degradation. When the pathway is inactive, dephosphorylated YAP/TAZ translocate into the nucleus, bind to the TEAD transcription factors, and initiate the expression of pro-oncogenic genes. As indicated, lncRNA SNHG3 regulates key components of this pathway by sequestering miR-630. **b** **Wnt/β-Catenin Signaling Pathway:** In the absence of Wnt signaling, cytoplasmic β-catenin is bound by the “destruction complex” comprising APC, Axin, GSK-3β, and CK1α, leading to its phosphorylation and ubiquitin-mediated degradation. Upon Wnt activation, signaling through Disheveled (Dsh) inhibits the destruction complex, allowing stabilized β-catenin to accumulate in the nucleus. Nuclear β-catenin binds to TCF/LEF transcription factors to drive target gene transcription. Multiple lncRNAs regulate this process: for instance, HNRNPU-AS1 influences pathway components by sponging miR-485-3p; the SPINT1-AS1/miR-214 axis, the CDKN2B-AS1/miR-665 axis, and the DANCR/miR-665 axis have all been shown to regulate β-catenin stability and/or nuclear translocation. UCK2 is also implicated as a target in this context. **c** **TGF-β** **Signaling Pathway:** Binding of TGF-β ligands to their receptors leads to the phosphorylation of receptor-regulated SMADs (R-SMADs: SMAD2/3). Phosphorylated R-SMADs then form a complex with the common mediator SMAD4 (Co-SMAD), which translocates to the nucleus to regulate gene expression. The inhibitory SMAD7 provides negative feedback. The lncRNA DANCR, via its interaction with miR-665, is involved in modulating this pathway, potentially affecting elements such as SARA. In summary, these lncRNAs function as critical molecular “sponges” through the ceRNA mechanism, exerting precise control over the activity of the aforementioned oncogenic signaling pathways and significantly influencing cervical cancer progression. APC Adenomatous Polyposis Coli, Axin Axis Inhibition Protein, CKIα Casein Kinase I alpha, FAT1-4 Fatty Acid Transport Protein 1-4, FRMD6 FERM Domain Containing 6, GSK-3β Glycogen Synthase Kinase-3 beta, LATS1/2 Large Tumor Suppressor Kinase 1/2, LRP Low-Density Lipoprotein Receptor-Related Protein, MST1/2 Macrophage Stimulating 1/2, MOB1 Mps One Binder Kinase Activator 1, SARA SMAD Anchor for Receptor Activation, SAV1 Salvador Family WW Domain Containing Protein 1, SMAD2/3 Mothers Against Decapentaplegic Homolog 2/3, SMAD4 Mothers Against Decapentaplegic Homolog 4, SMAD7 Mothers Against Decapentaplegic Homolog 7.TCF/LEF, T-cell Factor/Lymphoid Enhancer Factor, TEAD Transcriptional Enhanced Associate Domain, TGF-β Transforming Growth Factor-beta.

LncRNAs significantly affect tumor progression by activating or inhibiting specific signaling pathways, thereby regulating cell growth, survival, and migration [35]. For instance, the Wnt signaling pathway exerts pivotal control over cell proliferation, differentiation, and tumorigenesis [36]. Several studies indicate that LncRNAs, including SPINT1-AS1, HNRNPU-AS1, LINC00665, EGFR-AS1, and SAHG6 promote CC proliferation and invasion via the Wnt signaling pathway [37–41]. The MAPK pathway play crucial roles in cell migration, proliferation, and apoptosis [42]. By negatively regulating the MAPK pathway, LncRNA CASC2 suppresses both the proliferation and migration of cervical cancer cells [35]. The therapeutic potential of targeting the MAPK pathway in various cancers is highlighted by small-molecule inhibitors like celastrol, which suppresses the HSDL2/MAPK/ERK signaling cascade to trigger apoptosis in breast cancer cells [43]. In contrast, the interaction of LncRNA LINC00997 with miR-574-3p activates the MAPK pathway-associated protein CUL2, thereby promoting the proliferation, migration, and invasion of cervical cancer cells [44]. These studies highlight the complex regulator mechanisms of LncRNAs in CC and offer potential directions for targeted therapy (Fig. 2).

LncRNAs finely regulate gene expression through DNA methylation, histone modification, and interactions with transcription factors, thereby affecting CC cell proliferation. For example, LncRNA HOTAIR promotes cell proliferation by recruiting the PRC2 complex, enhancing histone H3K27 trimethylation, and inhibiting tumor suppressor gene expression [45, 46]. In contrast, LncRNA GAS5 inhibits CC cell proliferation by interacting with METTL3 and reducing ALKBH5-mediated m6A modifications [47]. Furthermore, LncRNA TUG1 significantly promotes CC cell proliferation by regulating the activity of the transcription factor E2F1 [48]. These findings reveal a complex regulatory network of LncRNAs in CC cell proliferation, highlighting their diverse mechanisms of action in tumor progression.

### Regulation of apoptosis in CC cells

Research in cervical cancer has identified LncRNAs as critical modulators of apoptosis. As key molecular regulators, LncRNAs influence the survival and death of CC cells by affecting apoptosis-related gene expression, activating signaling pathways, and regulating cell cycle progression through various mechanisms.

A common oncogenic strategy involves apoptosis suppression, which several LncRNAs achieve by targeting core apoptotic regulators. Research indicates that specific LncRNAs suppress apoptosis by modulating the expression of both anti-apoptotic genes, such as Bcl-2, and pro-apoptotic genes, like p53. For instance, by sequestering miR-678-5p, LncRNA MNX1-AS1 elevates Bcl-2 expression, an action that subsequently promotes the proliferation, migration, and invasion of cervical cancer cells [49]. Similarly, LncRNA CCAT2 expression is significantly elevated in CC tissues, thereby promoting cell proliferation by inhibiting p53 expression and further exacerbating tumor growth and spread [50]. These examples demonstrate a convergent mechanism in which different LncRNAs disable the apoptotic machinery by targeting distinct but crucial nodes such as the Bcl-2 family, p53, or mTOR pathway.

In contrast to these oncogenic LncRNAs, some LncRNAs function as tumor suppressors by actively promoting apoptosis. A prime example of this is PTCSC3. Downregulation of LncRNA PTCSC3 is closely linked to CC cell apoptosis, whereas its overexpression significantly inhibits proliferation, induces cell cycle arrest, and promotes tumor growth inhibition by driving cells to the apoptotic stage [51]. These findings underscore the intricate regulatory mechanisms by which LncRNAs modulate apoptosis in cervical cancer cells.

Collectively, these findings reveal a critical dichotomy in apoptosis regulation by LncRNAs. These studies not only reveal the critical role of LncRNAs in regulating cell proliferation but also highlight their involvement in apoptosis-related signaling pathways, showcasing their diverse regulatory functions in tumor biology. This study provides a new molecular perspective on CC development and progression.

### Functions of LncRNAs in CC cell invasion and metastasis

Invasiveness and metastatic ability are critical factors that influence the prognosis of patients with CC, and are closely linked with disease progression and recurrence. Studies have shown that patients with early invasive CC have a significantly lower 5-year survival rate than those without invasion. Additionally, lymph node and distant metastases are key indicators of poor prognosis in patients with CC [52, 53]. In this process, LncRNAs play a crucial role by orchestrating a multipronged attack that involves rewiring the internal machinery of cancer cells and remodeling their external environment, making them important molecular targets for treatment.

Primarily, LncRNAs directly augment the intrinsic migratory and invasive potential of cervical cancer cells through molecular mechanisms such as functioning as competing endogenous RNAs (ceRNAs) or activating pro-metastatic signaling pathways. Dong et al. demonstrated that LncRNA SNHG12 was significantly upregulated in CC tissues and correlated with FIGO stage and lymph node metastasis. SNHG12 knockdown reduces CC cell migration and invasion by regulating miR-424-5p expression [54]. The oncogenic factor LncRNA AC010883.5, which is overexpressed in CC tissues and cell lines, enhances cellular migration and invasion. This is achieved by activating the MAPK signaling pathway via the increased phosphorylation of ERK1/2 and MEK1/2, thereby promoting CC progression [55]. Additionally, LncRNA LINC00319 significantly promotes CC cell migration and invasion by regulating the miR-3127-5p/RPP25 axis [56]. These examples illustrate how different LncRNAs enable individual cancer cells to move and invade through distinct molecular routes.

**Fig. 3:**
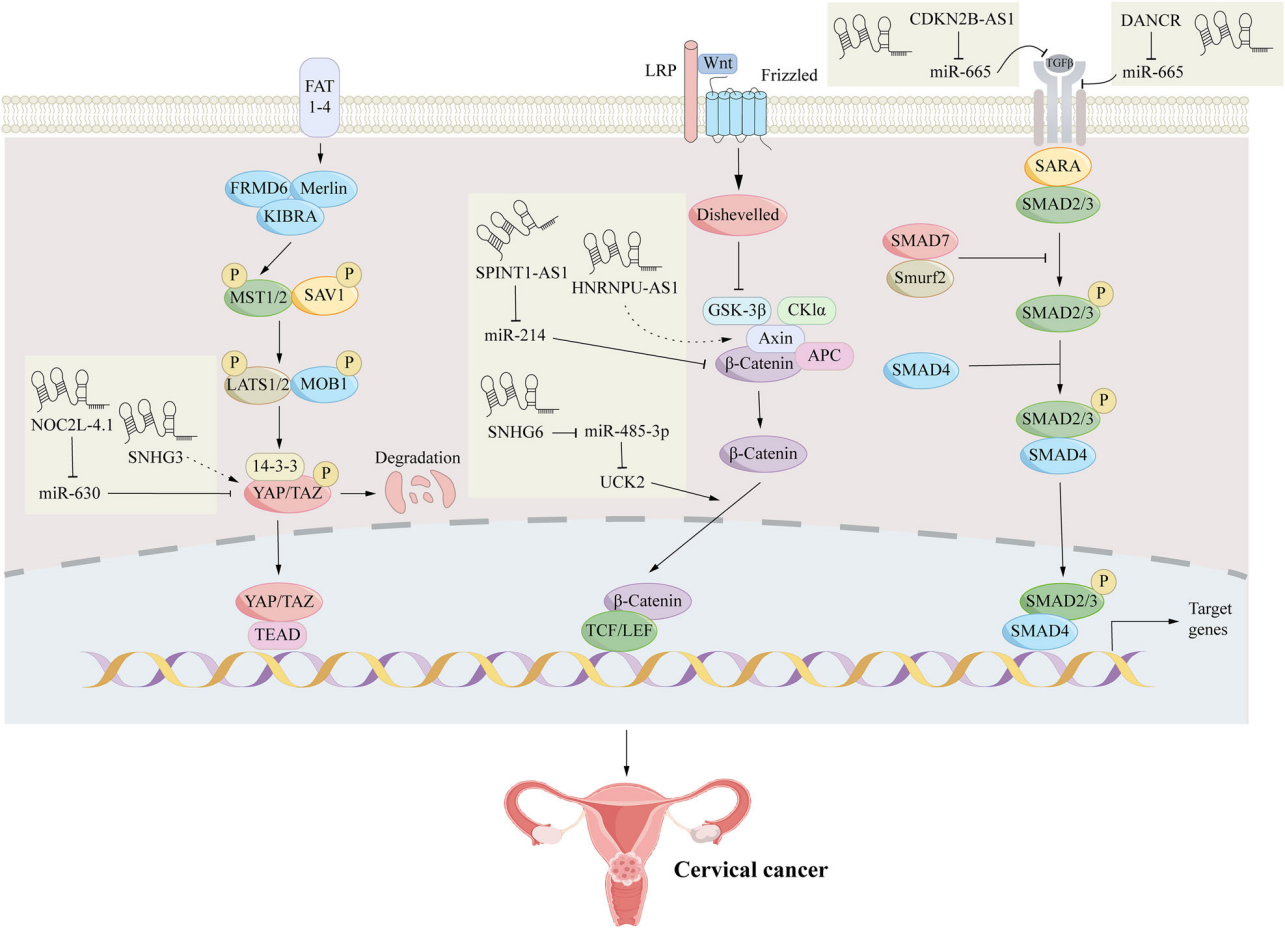
Long non-coding RNAs (lncRNAs) and how it interacts with tumor surroundings. In breast cancer, the Nuclear Factor Kappa B Interacting Long Noncoding RNA (NKILA) acts as a tumor suppressor. It effectively reduces breast cancer cell proliferation, epithelial-mesenchymal transition (EMT), and metastatic potential by directly inhibiting the NF-κB signaling pathway. Furthermore, the function of NKILA extends to immune cells within the tumor microenvironment: in tumor-infiltrating T lymphocytes, NKILA enhances sensitivity to activation-induced cell death (AICD) by modulating the NF-κB pathway, which may paradoxically dampen anti-tumor immune responses, highlighting the complexity of its role. Hypoxic conditions within the tumor microenvironment activate Calcium/calmodulin-dependent protein kinase A (CamK-A), which in turn activates the NF-κB pathway. This activation leads to the upregulation of pro-inflammatory and pro-angiogenic factors such as Interleukin-6 (IL-6), IL-8, and Vascular Endothelial Growth Factor (VEGF). Consequently, this promotes the recruitment of tumor-associated macrophages and tumor angiogenesis, supporting tumor growth and metastasis. In contrast, in bladder cancer, Lymph Node Metastasis-Associated Transcript 1 (LNMAT1) exerts an oncogenic function. LNMAT1 specifically activates the transcription of the chemokine CCL2 by interacting with its binding partner, heterogeneous nuclear ribonucleoprotein L (hnRNPL). The secretion of CCL2 then recruits macrophages to the tumor site, remodeling the tumor microenvironment and ultimately driving lymphatic metastasis in bladder cancer.

In addition to single-gene regulation, LncRNAs can orchestrate the entire epithelial-mesenchymal transition (EMT), a fundamental phenotypic switch required for metastasis [57, 58]. In cervical cancer, LncRNAs drive tumor cell migration and invasion by governing the expression of genes linked to the EMT. A key example is the highly expressed LncRNA SNHG7, which orchestrates the EMT program by suppressing E-cadherin while elevating the levels of N-cadherin and vimentin [59, 60]. Likewise, LncRNA TP73-AS1 correlates with a poor prognosis by driving cervical cancer cell migration and invasion through the regulation of EMT markers [61]. LncRNAs can also function as ceRNAs by competitively binding to miRNAs to release repressed mRNAs and regulate EMT-associated genes. LINC01305 promotes EMT in CC cells [62]. Collectively, these findings demonstrate that by modulating the expression of EMT-related genes, LncRNAs drive the phenotypic transitions that promote invasion and metastasis in cervical cancer, thereby establishing their significance in tumor progression (Fig. 3).

Second, in addition to altering cancer cells themselves, LncRNAs promote metastasis by actively remodeling the extrinsic tumor microenvironment (TME), thereby creating a permissive escape path [63]. By modulating immune cell function and the metabolic state of tumor cells, LncRNAs are capable of remodeling the TME. For example, LncRNA HULC enhances cervical cancer cell proliferation and invasion by functioning as a sponge for miR-218, a process that simultaneously contributes to the regulation of immune responses within the TME [64]. Additionally, LncRNA ATB regulates miR-144 and ITGA6 expression, promoting tumor cell proliferation and invasion, highlighting its crucial role in the TME [65].

Extracellular matrix (ECM) remodeling is a key process in tumor cell invasion and metastasis, and it involves the degradation and rearrangement of ECM components [66]. Several studies have shown that LncRNAs regulate ECM-related gene expression by interacting with molecules such as miRNAs, proteins, and chromatin. For example, LncRNA ANRIL promotes CC cell invasion and metastasis by interacting with the PRC2 complex and regulating the ECM signaling network [67]. Additionally, some LncRNAs promote ECM degradation and rearrangement by upregulating or downregulating specific ECM-related genes (e.g., types I and II collagen), providing space for tumor cell invasion and metastasis [68]. Thus, LncRNAs not only provide cancer cells with the tools to move but also physically clear the path for their escape by dismantling surrounding tissue barriers.

These findings reveal the versatile role of LncRNAs in the TME, which promote CC invasion and metastasis by precisely regulating immune cell function, tumor cell metabolic status, and remodeling the ECM signaling network. Elucidation of these mechanisms highlights the central role of LncRNAs in CC progression and provides a solid theoretical foundation for LncRNA-based targeted therapies, potentially opening new avenues for the precise treatment of CC.

## Clinical significance of LncRNAs in CC

### LncRNAs as diagnostic markers for CC

The 5-year survival rate for early-stage CC (e.g., stages 0 and IA) exceeds 90%, whereas it ranges between 36–53% in advanced stages (III and IV) [69, 70].Thus, enhancing the screening and early diagnosis of CC is crucial. However, current screening methods have significant limitations. Currently, early diagnosis and screening of CC mainly depend on HR-HPV detection and cytology (ThinPrep cytologic test [TCT]) [71]. The HR-HPV test has high sensitivity but low specificity, leading to false positives that may lead to overdiagnosis and unnecessary further testing or treatment. Although the TCT can improve detection rates, its false-negative rate may still lead to missed diagnoses [72]. Combined testing reduces missed diagnoses and misdiagnoses, but significantly increases screening costs and complexity. Moreover, the low specificity of HR-HPV testing can lead to high false positive rates. Inconsistent HR-HPV and TCT results require further colposcopy, thereby increasing medical resource consumption and reducing patient compliance [73].

As studies progress, the expression of multiple LncRNAs in CC has shown significant diagnostic value, with levels significantly higher than those in normal tissues, making them potential biomarkers. For example, Yan et al. found that HOTAIR levels in vaginal secretions and serum of patients with CC were higher than those in a normal population, positively correlating with tumor malignancy and showing higher diagnostic performance in vaginal secretions (89.3% vs. 92.7%). Three months postoperatively, HOTAIR expression in vaginal secretions and serum was significantly decreased, highlighting its role as an important biomarker for CC diagnosis and prognosis [74].

Experimental knockdown of LncRNA BLACAT1 impeded cervical cancer progression by inducing G0/G1 cell cycle arrest, which curtailed proliferation, and by markedly impairing the migratory and invasive capacities of ME180 and C33A cells. These findings underscore the oncogenic function of BLACAT1, positioning it as a promising candidate for both clinical diagnosis and therapeutic intervention [75]. Quantifying the expression of these LncRNAs could pave the way for novel non-invasive methods for the early detection of cervical cancer.

Additionally, the detection of circulating LncRNAs is considered a promising method for early diagnosis because of their stability in blood and ease of collection and analysis, making them ideal biomarkers [76]. Specific circulating LncRNAs have been significantly upregulated in the plasma of CC patients, offering new possibilities for early diagnosis [77]. Specific circulating LncRNAs are significantly upregulated in the plasma of patients with CC, offering new possibilities for early diagnosis [77]. Liquid biopsy techniques allow physicians to monitor tumor markers without invasive surgery, thereby enabling early detection and dynamic monitoring [78]. This method improves diagnostic accuracy while providing a safer and more convenient examination for patients.

In summary, numerous studies have shown significant changes in the expression patterns of multiple LncRNAs in patients with CC, emphasizing their key roles in CC biology and highlighting their potential as diagnostic markers. Therefore, an in-depth exploration of LncRNA function and its regulatory mechanisms in CC is expected to provide new strategies and targets for early diagnosis, targeted treatment, and prognosis monitoring.

### Progress in LncRNAs as therapeutic targets for CC

Although significant progress has been made in the treatment of CC, particularly in terms of surgical resection, radiotherapy, chemotherapy, targeted therapy, and immunotherapy, the survival rate of patients with advanced CC remains low. This is primarily because of the complexity of advanced CC and limitations of treatment options [79]. Additionally, patients with advanced CC often have metastasis or recurrence, further complicating treatment and increasing the risk of poor prognosis [80]. Recently, significant progress has been made in the study of LncRNAs in CC treatment. Various LncRNAs play crucial roles in the occurrence, development, and prognosis of CC, and are expected to serve as novel therapeutic targets [81].

Several therapeutic strategies have been developed to target oncogenic LncRNAs, which are broadly categorized by their mode of action: directly modulating LncRNA levels, interfering with their molecular interactions, or improving the delivery of therapeutic agents. The most direct approach is to reduce the expression or function of oncogenic LncRNAs by using various molecular tools.

Small molecule inhibitors can block the effects of LncRNAs in CC by directly targeting their expression or function. For example, small molecule inhibitors targeting LncRNAs, such as LINC01535 and HOTAIR, effectively inhibit the proliferation, migration, and invasion of CC cells. Although studies in this field is still in its early stages and further optimization of drug design is required to enhance specificity and efficacy, these preliminary findings provide strong theoretical support for LncRNA-based targeted therapy, suggesting that it may be a promising direction for CC treatment [82].

**Fig. 4:**
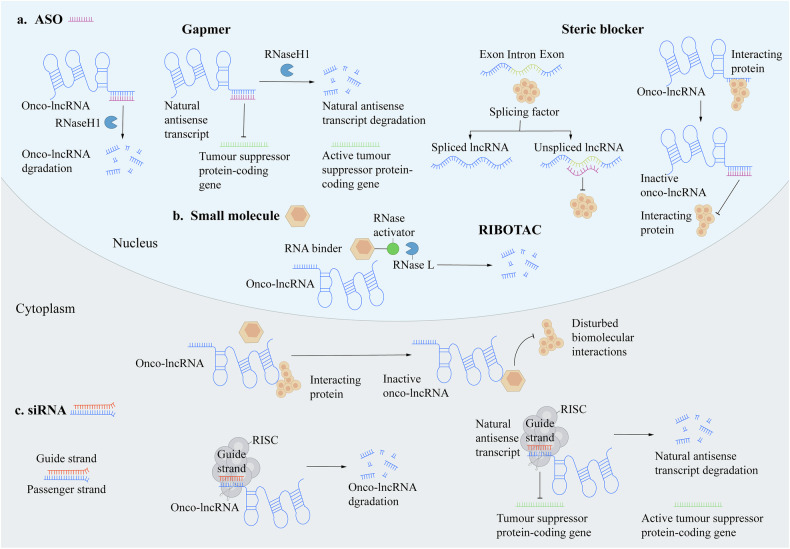
There are three main therapeutic strategies for the loss of function of long non-coding RNAs (lncRNAs). **a** Antisense Oligonucleotides (ASOs).**a** ASOs, such as gapmers, are single-stranded nucleic acids designed to be complementary to a specific onco-lncRNA sequence. Upon binding to their target lncRNA in the nucleus, they form a DNA-RNA heteroduplex. This duplex is recognized and cleaved by the cellular enzyme RNase H1, leading to the degradation of the lncRNA. A key application is targeting natural antisense transcripts (NATs), which often repress tumor suppressor genes. Degrading the repressive NAT allows for the reactivation of the adjacent tumor suppressor protein-coding gene. **b** Small Molecules. Small molecules can interfere with lncRNA function through multiple mechanisms. They can act as RNA binders that disrupt the lncRNA’s secondary structure, which is crucial for its activity. Alternatively, they can function as structural blockers, preventing the lncRNA from adopting its active conformation. A more sophisticated approach involves molecules that disrupt the interaction between the lncRNA and its essential protein partners (interacting proteins), thereby inhibiting the formation of functional complexes. Furthermore, RIBOTACs (Ribonuclease-Targeting Chimeras) are bifunctional small molecules that bind simultaneously to a specific lncRNA and recruit cellular ribonucleases (RNases) to directly degrade the target. **c** Small Interfering RNAs (siRNAs). siRNAs are synthetic double-stranded RNA molecules. Upon entering the cell, the passenger strand is discarded, and the guide strand is loaded into the RNA-induced silencing complex (RISC). The guide strand then directs RISC to the complementary sequence of the target lncRNA, leading to its precise cleavage and degradation. Like ASOs, siRNAs can be used to degrade repressive natural antisense transcripts, thereby promoting the expression of tumor suppressor genes.In summary, these strategies—ASOs, small molecules, and siRNAs—offer diverse avenues to silence oncogenic lncRNAs by degrading the transcript itself or disrupting its functional interactions, providing promising therapeutic potential for cancer treatment.

Similarly, RNA interference technologies, such as small interfering RNA (siRNA) and antisense oligonucleotides (ASOs) exert therapeutic effects by specifically inhibiting the expression of target LncRNAs. For example, targeted inhibition of LncRNA LINC01535 using siRNA significantly reduces the proliferation and metastasis of CC cells. Additionally, ASOs specifically target and suppress LncRNA expression through RNase-mediated degradation, reducing the malignant phenotype of CC cells [82–84]. CRISPR/Cas9 technology is an efficient gene-editing tool that enables the direct targeted deletion of specific LncRNA genes. Knockdown of oncogenic LncRNAs (e.g., HOTAIR) in CC cells effectively prevents cancer cell proliferation and invasion. Although challenging in clinical applications, its precision and efficiency make it a promising candidate for CC treatment [84–86](Fig. 4).

An alternative indirect strategy is to disrupt the functional complexes that LncRNAs form with other molecules, such as RNA-binding proteins (RBPs). Certain LncRNAs promote the development and progression of CC by interacting with RNA-binding proteins to form functional complexes that regulate gene expression, chromatin remodeling, or signaling pathway activation. As a case in point, the oncogenic LncRNA EBIC facilitates the invasion of cervical cancer cells by interacting with EZH2, which in turn suppresses the expression of E-cadherin [87]. Through its interaction with YBX1, HOTAIR facilitates its own nuclear translocation while concurrently modulating cell proliferation via the PI3K/Akt and ERK/RSK signaling pathways [88]. By interfering with the functions of these RBPs, the effects of LncRNAs can be indirectly inhibited, thereby achieving therapeutic effects. This strategy offers a new approach for CC treatment.

Finally, a critical component for the clinical success of any LncRNA-targeted therapy is the development of efficient drug delivery systems, such as nanoparticles, liposomes, and viral vectors, that can effectively deliver therapeutic molecules into CC cells. This improves the accumulation of therapeutic molecules in CC tissues, enhances therapeutic efficacy, and reduces drug damage to healthy cells, thereby minimizing side effects [82, 89]. This will significantly advance the precision and personalized development of CC treatment, thereby providing patients with more effective and safer options. In the future, as these delivery technologies are continuously optimized and translated into clinical practice, therapies targeting LncRNAs are expected to play a larger role in treating malignancies, such as CC, and may even become breakthrough treatments.

### LncRNAs in Chemotherapy resistance of CC

Chemotherapy is a mainstay treatment for locally advanced CC, as demonstrated in several studies. Cisplatin-based regimens are commonly used [90, 91]. Cisplatin-based regimens are commonly used; however, their efficacy is frequently limited chemoresistance, which is a major clinical challenge that contributes to treatment failure and poor prognosis. Emerging evidence indicates that LncRNAs are pivotal players in orchestrating the molecular pathways that drive this resistance [92].

The expression of LncRNAs in cervical cancer is a critical determinant of chemoresistance. The aberrant expression of multiple LncRNAs has been observed in drug-resistant gliomas and their related cell lines [93]. Oncogenic LncRNAs often promote resistance through a variety of interconnected mechanisms, including inducing EMT, acting as miRNA sponges to derepress drug resistance genes, and modulating key survival pathways. For instance, LncRNA HOTAIR has been shown to promote chemoresistance in cervical cancer by inducing the EMT and modulating the miR-29b/PTEN/PI3K pathway. Mechanistically, it functions as a molecular sponge for miR-29b, thereby diminishing cellular sensitivity to agents like cisplatin, paclitaxel, and docetaxel [94].

Likewise, LncRNA PVT1 is deeply implicated in the tumorigenesis and progression of numerous human cancers and may also enhance cellular sensitivity to both chemotherapy and radiotherapy [95, 96]. In cervical cancer, elevated LncRNA PVT1 expression correlates with poor prognosis and an aggressive phenotype, driving chemoresistance by sequestering miR-195 to regulate the epithelial-mesenchymal transition (EMT)—a mechanism induced by hypoxia and immune responses. Conversely, silencing PVT1 suppresses cell proliferation, invasion, and migration while enhancing sensitivity to cisplatin [97, 98]. Importantly, LncRNA PVT1 blocks paclitaxel-induced EMT and sensitizes CC cells to paclitaxel treatment. These findings suggest that PVT1 influences chemoresistance in CC cells by regulating miR-195 and EMT, indicating that LncRNA PVT1 could be a key target in overcoming chemoresistance [98].

LncRNA UCA1 is deeply implicated in the processes of tumorigenesis and malignant progression [99, 100]. Studies have shown that LncRNA UCA1 upregulation enhances cisplatin resistance, promotes cell proliferation, and reduces apoptosis in CC cells. In contrast, UCA1 downregulation reduces cisplatin resistance in CC cells. Therefore, blocking UCA1 expression could be an effective strategy for treating CC, thereby helping to overcome cisplatin resistance and inhibit tumor progression [101].

Conversely, some tumor-suppressive LncRNAs can sensitize CC cells for chemotherapy, and their downregulation is often associated with acquired resistance. In cervical cancer, the established tumor suppressor LncRNA GAS5 mediates its effects by targeting miR-21 [102, 103]. Overexpression of LncRNA GAS5 suppresses the proliferation, invasion, and migration of cervical cancer cells while simultaneously enhancing the sensitivity of chemoresistant cells to cisplatin. This is achieved by inhibiting miR-21, which leads to the upregulation of PTEN, subsequent attenuation of the pAkt signaling pathway, and a consequent reduction in cisplatin resistance [104]. In patients with cervical cancer, diminished LncRNA GAS5 expression is a key indicator of both cisplatin resistance and poor survival [105]. Consequently, LncRNA GAS5 emerges as a compelling therapeutic target for overcoming cisplatin resistance in CC.

In conclusion, LncRNAs are pivotal mediators of therapeutic resistance in cervical cancer, positioning them as compelling targets for strategies aimed at overcoming drug resistance and improving patient outcomes. Although chemotherapy is the conventional treatment for CC, its efficacy is limited by drug resistance. Therefore, targeting LncRNAs may offer an effective strategy for reversing drug resistance and enhancing treatment outcomes, thereby providing new avenues for clinical treatment.

### LncRNAs in prognostic assessment of CC

LncRNAs are recognized as significant players in the diagnosis, treatment, and prognosis of cervical cancer (CC). A pertinent example is LncRNA LINC00675, which Ma et al. reported is elevated in CC tissues and cell lines, where its expression corresponds with advanced clinical stage and unfavorable patient outcomes. In line with these clinical observations, in vivo experiments confirmed that silencing LINC00675 inhibited tumor growth. This suppressive effect was accompanied by significant molecular alterations within the tumor tissue, including the upregulation of the pro-apoptotic proteins Bax and GSK-3β and the downregulation of the anti-apoptotic and oncogenic proteins Bcl-2 and β-catenin. Supporting this clinical correlation, mechanistic studies have revealed that LINC00675 actively drives tumor growth in vivo. This is accomplished by its capacity to fundamentally dysregulate a network of molecular pathways governing cell proliferation and survival, a potent biological function that directly explains why its expression serves as a key determinant of adverse patient outcomes [106].

In CC, miR-214 has been demonstrated to have a tumor-suppressive effect, and its expression level is closely related to patient prognosis [107, 108]. Son et al. have elucidated a mechanism whereby EZH2 directly inhibits the tumor-suppressive function of miR-214. They found that LncRNA LINC01535 expression is positively correlated with that of EZH2, suggesting LINC01535 may influence miR-214 expression by regulating EZH2. Consistent with this, functional assays revealed that elevated LINC01535 expression promotes the growth, migration, and invasion of cervical cancer (CC) cells in vitro and enhances xenograft tumor growth in vivo. Clinically, LINC01535 is significantly upregulated in CC tissues and is strongly associated with advanced clinical stages and poor prognosis.

Collectively, these demonstrated oncogenic functions provide the crucial biological basis for the observed clinical association, establishing a direct and compelling mechanistic link between the molecular activities of LINC01535 and its role as a powerful indicator of advanced disease stage and poor patient prognosis [93].

Additionally, Liu et al. found that LncRNA LINC00861 expression was significantly downregulated in CC tissues and in patients with CC having advanced lymph node metastasis, which correlated with poor prognosis. Providing a clear mechanistic rationale for this clinical observation, functional studies confirmed that overexpressing LINC00861 potently suppresses key hallmarks of malignancy, including the proliferation, migration, invasion, and the epithelial-mesenchymal transition (EMT) of CC cells, thereby highlighting its crucial protective role that directly contributes to better patient outcomes [109]. Other studies have shown that the expression levels of LncRNAs TP73-AS1 and SPINT1-AS1 are closely linked to patient prognosis. High expression of TP73-AS1 is considered an independent factor for poor prognosis in patients with CC and is correlated with tumor aggressiveness and metastasis [110, 111].

More importantly, establishing LncRNA-based prognostic models enables physicians to better assess patient survival and treatment response, thereby facilitating the development of personalized treatment plans. LncRNA-based prognostic models have been used to predict the survival of patients with CC. For example, Zhong et al. found that specific LncRNAs were significantly associated with metabolic, immune, and other CC pathways by integrating DNA methylation, transcriptome data, and copy number variations to successfully construct a prediction model. These models demonstrated stable predictive performance in both training and testing sets, offering new biomarkers for the prognostic management of patients with CC [112].

Additionally, Chen et al. predicted CC prognosis by analyzing the six-LncRNA immune prognostic signature constructed by their team using receiver operating curve-area under the curve. The Kaplan–Meier analysis showed that patients with higher risk scores had a worse prognosis, a conclusion that was consistently validated in various subgroup analyses. Moreover, the risk score is closely linked to the clinicopathological characteristics and tumor grade of patients and can serve as an independent risk factor for CC prognosis [113].

Dysregulated fatty acid metabolism (FAM) is a pivotal driver of progression in various tumors [114, 115]. In an effort to assess the prognostic relevance of fatty acid metabolism (FAM) in cervical cancer, Lang et al. constructed a predictive model based on a nine-LncRNA signature. This signature effectively stratified patients, revealing that those in the high-risk cohort experienced significantly shorter overall survival. With its validation as an independent prognostic factor by multivariate analysis, this nine-LncRNA panel emerges as a highly promising biomarker for forecasting patient outcomes and informing the clinical deployment of FAM-targeted therapies [116].

In summary, LncRNAs have a significant potential for application in the prognostic evaluation of CC. By constructing LncRNA-based prognostic models, more accurate survival predictions and personalized treatment strategies can be provided. However, this study requires further validation and refinement before it can be widely applied in clinical practice.

## Future research directions of LncRNA in CC

### In-depth exploration of LncRNAs’ regulatory mechanisms in CC

As a critical class of regulatory molecules, LncRNAs are increasingly recognized for their integral function in the onset, progression, and drug resistance of CC. They contribute to the complex regulatory network of CC through multifaceted mechanisms, including the modulation of gene expression, epigenetic alterations, the remodeling of signaling pathways, and immune regulation. The interplay among these processes ultimately dictates the phenotype of CC cells, influencing tumor development, progression, and therapeutic response.

Although the fundamental contribution of LncRNAs to the initiation, progression, and drug resistance of cervical cancer is well-documented, the intricate regulatory networks they orchestrate through molecular interactions remain incompletely understood [117]. LncRNAs not only function at the gene transcription level but also regulate various biological processes in CC by influencing other molecular mechanisms within the cell [118]. Therefore, elucidating the specific interactions between LncRNAs and other molecules—such as miRNAs, transcription factors, and chromatin modifiers—is essential for uncovering the molecular foundations of cervical cancer and paving the way for novel diagnostic and targeted therapeutic strategies.

In the future, studies targeting LncRNAs should focus on revealing their specific mechanisms of action in CC and exploring their complex interactions with various molecules in the cell. This will not only improve our understanding of the molecular regulatory network of CC but also provide a breakthrough in developing new diagnostic markers and screening therapeutic targets. Through systematic studies, LncRNAs are expected to become key targets in precision medicine and individualized treatment for CC, offering new perspectives for clinical treatment [81]. Furthermore, investigating the potential for LncRNAs to modulate ferroptosis in cervical cancer, a process regulated by pathways like Nrf2-xCT/GPX4, offers a novel avenue for mechanistic research [119].

### Development of new LncRNA-based diagnostic and therapeutic strategies

With a deepening understanding of their regulatory mechanisms in CC, developing LncRNA-based diagnostic tools and therapeutic strategies is becoming a primary focus for future research. LncRNAs function as pivotal regulators in the development, progression, and drug resistance of CC by precisely modulating gene expression and signaling pathways through interactions with miRNAs, proteins, and other molecules [117]. Given the multidimensional regulation of LncRNAs in CC, future studies should focus on the development of novel LncRNA-based diagnostic tools and targeted therapies. These are expected to offer new approaches for early CC screening, advance personalized therapy, and provide patients with more accurate and effective treatment options, thereby significantly improving their survival rates and quality of life.

Specifically, the development of LncRNA-based diagnostic tools requires systematic identification and validation of clinically significant LncRNA markers that should enable accurate identification of patients with CC at early stages of the disease, thereby enabling early intervention. At the same time, the development of targeted therapy strategies based on LncRNAs should focus on exploring the regulatory role of LncRNAs in the TME, as well as their synergistic effects with existing treatments, to achieve more precise therapeutic effects. Additionally, interdisciplinary collaborations, including collaborative studies in areas, such as bioinformatics, molecular biology, and clinical medicine, will provide strong support for the development of LncRNA-based diagnostic and therapeutic strategies.

### Prospects of cross-omics studies in CC research

With the rapid developments in genomics, transcriptomics, proteomics, and metabolomics, the potential applications of these technologies in CC studies have grown significantly. The integration of multi-omics offers unprecedented opportunities to comprehensively analyze the multi-level molecular mechanisms of CC, particularly in exploring the regulatory role of LncRNAs [120, 121]. By enabling a systematic analysis of LncRNA expression profiles, mechanisms of action, and molecular interactions in cervical cancer (CC), cross-omics studies establish a theoretical foundation for novel diagnostic and therapeutic strategies. This comprehensive, multi-dimensional approach is essential for elucidating the intricate molecular landscape of CC, thereby providing the scientific rationale for developing personalized treatment plans aimed at improving patient outcomes.

## Challenges of LncRNAs in CC studies and clinical application

Although LncRNAs have shown significant potential in CC studies, they still face several challenges. First, the molecular mechanisms of LncRNAs are complex and unclear, particularly their specific roles in CC, including epigenetic regulation, post-transcriptional regulation, and interactions with molecules, such as mRNAs and miRNAs, which require further in-depth studies [50]. Second, technical challenges hinder the detection and functional studies of LncRNAs, particularly because of their low expression levels, poor stability, and safety and efficacy concerns with targeted delivery vectors [81]. Furthermore, the interaction of LncRNAs with the immune system and other biological processes remain unclear, hindering a comprehensive understanding of their roles [122]. Limited resources is another key factor limiting the progress of studies on LncRNA, particularly the lack of databases and analytical tools specifically designed for LncRNAs, which restricts the depth and scope of study [123].

Additionally, the clinical application of LncRNAs faces significant limitations, including lack of non-invasive detection methods and unclear relationships with drug resistance [124, 125]. The heterogeneity and limitations of the findings must also be addressed, particularly the lack of large-scale, multicenter clinical trial validation, as well as the considerable variations in LncRNA species and functions across studies [126, 127]. Finally, despite the potential of LncRNA-based therapeutic strategies, relevant therapeutic tools have not yet entered clinical trials [128, 129]. Therefore, although LncRNAs show great potential in CC studies, their complex molecular mechanisms, technical challenges, limitations in clinical applications, and study heterogeneity need to be addressed. In the future, extensive basic studies and clinical trials are needed to reveal the specific mechanisms of LncRNAs in CC and develop more effective detection and treatment strategies.

## Summary

Recent research underscores the integral role of LncRNAs in the development, progression, and clinical outcome of cervical cancer (CC). A growing body of evidence demonstrates that by modulating fundamental biological processes—including cell proliferation, migration, and apoptosis—LncRNAs are emerging as promising biomarkers for early diagnosis and as novel targets for individualized therapeutic strategies. These findings provide a new framework for understanding the molecular mechanisms of CC and establish a foundation for future clinical applications.

Thus, promoting the translation of LncRNAs into clinical applications in CC is a key goal for future studies. To achieve this goal, researchers must explore multiple aspects in depth. First, basic studies on LncRNA function must be conducted to clarify their specific role in CC and their interactions with other biomarkers. Second, translational studies must establish standardized assays to ensure the reliability and validity of LncRNAs in clinical applications. Additionally, the development of targeted LncRNA intervention strategies must be prioritized to provide more effective treatment options for patients with CC.

However, future studies should address several challenges. The diversity and complexity of LncRNAs make the elucidation of their functions challenging. Additionally, integrating LncRNA detection and application in clinical practice to ensure patient benefits remains a major challenge. Therefore, interdisciplinary collaboration and multicenter clinical trials are crucial for advancing LncRNA studies in the field of CC and facilitating its clinical translation.

In summary, the importance of LncRNAs in CC studies is undeniable, and future studies should offer deeper insights and new application opportunities. By systematically addressing the challenges in current studies, we can achieve significant breakthroughs in the prevention, diagnosis, and treatment of CC.
